# Opportunistic brood theft in the context of colony relocation in an Indian queenless ant

**DOI:** 10.1038/srep36166

**Published:** 2016-10-31

**Authors:** Bishwarup Paul, Manabi Paul, Sumana Annagiri

**Affiliations:** 1 Behaviour & Ecology Lab, Department of Biological Sciences, Indian Institute of Science Education and Research, Kolkata, Mohanpur, West Bengal 741246, India

## Abstract

Brood is a very valuable part of an ant colony and behaviours increasing its number with minimum investment is expected to be favoured by natural selection. Brood theft has been well documented in ants belonging to the subfamilies Myrmicinae and Formicinae. In this study we report opportunistic brood theft in the context of nest relocation in *Diacamma indicum,* belonging to the primitively eusocial subfamily Ponerinae. Pupae was the preferred stolen item both in laboratory conditions and in natural habitat and a small percentage of the members of a colony acting as thieves stole about 12% of the brood of the victim colony. Stolen brood were not consumed but became slaves. We propose a new dimension to the risks of relocation in the form of brood theft by conspecific neighbours and speculate that examination of this phenomenon in other primitively eusocial species will help understand the origin of brood theft in ants.

Offspring are very important for ensuring the survival of species, and thus caring for young is an important aspect of life; but the extent of this care is highly variable across the animal kingdom. Among mammals and birds caring for young is commonly seen in the form of parental care, and it is less common in reptiles, amphibians, fishes, arthropods, molluscs, annelids and other invertebrates^1^. Social insects, a subset of arthropods, have taken caring for young to the next level. In these societies, there are only one or a few reproductives laying eggs while other adults belonging to the worker caste tend to the needs of these reproductives and the offspring they produce^2^,^3^. The first three stages of the life cycle (eggs, larvae and pupae) of these holometabolous insects are collectively called brood, and is completely dependent on adults for their protection and care^4^. As the brood of a social insect colony represent the future workforce and reproductives, they are reared with care by the workers as their eclosion is essential for both the survival and reproduction of the colonies^3^,^5^.

In addition to representing the next generation, brood is known to play several different roles in the colonies of ants. Brood, especially larvae, act as the food reserve of a colony either by providing food to adults or by acting as food themselves. Adult-larva trophallaxis is a common phenomenon in social insects, where the adults provide food to the larvae, and the larvae exudate nutrient-rich liquid which the adult consumes^6^,^7^,^8^. For example, in the harvester ant species *Monomorium whitei* (*Chelaner whitei*) and *M. rothsteini* (*C. rothsteini*) the adults of the colony are fed by the larvae via trophallaxis^9^. The reproductives of an ant colony are generally fed by workers, but in some cases, like *Stigmatomma silvestrii* (*Amblyopone silvestrii*) and *Leptanilla japonica*, the primary source of queen nutrition is larval haemolymph. In these cases queens perform a non-destructive form of cannibalism whereby queens pierce the larvae and feed upon their haemolymph^10^,^11^. Cannibalism of all stages of eggs, larvae and pupae are also seen in ants. Damaged brood is promptly eaten, and during starvation the entire brood of a colony is gradually cannibalized^3^. Further, inter-specific brood predation where ants raid nests of other species to obtain brood for consumption has been documented^8^. Army ants are especially noted for their raiding behaviour, where they form specialized columns or swarm and hunt for food, which includes brood of other ants^3^,^12^. In a study on the army ant *Neivamyrmex rugulosus*, the authors observed that raided nests of fungus-growing ant *Trachymyrmex arizonensis* were left only with 25% of their brood^13^.

The procured brood item, especially pupae, has greater utility than serving only nutritional needs. As it is the last developmental stage and ecloses into adult ants, it has the potential of joining the workforce of the colony. Thus, incipient colonies steal brood from mature ones during colony foundation to quickly increase their workforce and the probability of survival. This has been recorded in four species of ants - *Myrmecocystus mimicus*, *Solenopsis invicta*, *Veromessor pergandei* and *Acromyrmex versicolor*^14^. Furthermore, species known as “slave-maker” or “dulotic” ants raid nests of other species to obtain pupae. These raided pupae, after eclosing into adults, start working as slaves for the raiding colony. In most cases dulosis is obligatory, i.e. the slaves do all the work for the colony, and the workers of the slave-maker are only specialized in fighting and raiding for slaves. Some species of *Polyergus*, *Strongylognathus*, *Harpagoxenus*, *Chalepoxenus* etc. are examples of such dulotic ants. However, a few facultative dulotic species are also known, and the adults of these species are capable of conducting all the tasks of the colony and occasionally conduct raids to utilise the services of the slaves, e.g. *Formica sanguinea*^8^,^15^.

The term ‘theft’ has anthropomorphic implications, but looking beyond these implications we find that the phenomenon is common in the animal kingdom and is present in several taxa starting from invertebrates to higher mammals. While theft of food is common, other objects such as nest material and brood are also stolen^16^,^17^. Among ants thievery of brood in the contexts of slave-making, predation and colony foundation has been predominantly seen in subfamilies Formicinae and Myrmicinae^15^, whereas there are no records in Ponerinae. In this study we explore opportunistic theft of conspecific brood in *Diacamma indicum*, a primitively eusocial ant belonging to the subfamily Ponerinae. This species is found in southern and northern India, Sri Lanka and Japan. Colony size is not large and ranges from 12–261 adults and the colonies are prone to emigrate with slight physical disturbance to their nests^18^,^19^. Nests of *Diacamma* spp. are monodomous^20^. *D. indicum* typically nests underground, but they have opportunistic nesting habit and have been seen to nest under stones, in cracks of walls and brick piles, in tree branches and trunks, in fallen logs and other sites^18^,^21^. The colonies are queenless, instead a single worker mates and reproduces, who is known as the gamergate^20^,^22^. Like many other genera of the subfamily Ponerinae, *D. indicum* also shows tandem running for colony relocation^8^ and it has previously been reported to be the only means used to recruit nestmates to the new nest^23^,^24^. During relocation in nature, colony members took shelter in 1–8 temporary nest sites (on average 4 sites) before they merged into a single final nest. The final nest was 1.4 m away from the old nest on average, and the relocations took on average 385 minutes^24^. Unlike some social insects like honeybees and wasps, ants move brood and stored resources in addition to adult members when they relocate from their old nest to a new nest. During relocation, colonies are particularly vulnerable to predation and exposure to harsh environmental condition as their nest no longer offers protection. In addition the adults who guard these resources would be occupied with other tasks related to relocation like searching for new nests or recruiting colony members, making the brood and any stored resources even more vulnerable. Thus during relocation several factors are expected to compromise the defence of the colony.

In this study we have investigated if colonies that are in the process of relocation face an additional threat in the form of neighbouring conspecific colonies. The first observation of *D. indicum* procuring conspecific brood was made during an unrelated study on vulnerability of brood in the natural habitat, where *D. indicum* larvae and pupae were kept unguarded. It was observed that several species of ants, along with *D. indicum*, attacked and carried away brood items^19^. We wanted to investigate whether adults from neighbouring conspecific colonies take advantage of the vulnerability of relocating colonies. We specifically asked if *D. indicum* workers steal brood, and further we examined the type of brood that is stolen and the fate of the procured brood in the thieving colony.

## Results

In this section we report our findings organized into different themes. We quantify the attempts of theft and the risk of brood theft in the laboratory based experiment. Then we compare direct observation of theft and indirect evidence of theft in the natural habitat. Lastly we examine the outcome of procured foreign brood in colonies.

### Brood theft in laboratory

**Figure 1:**
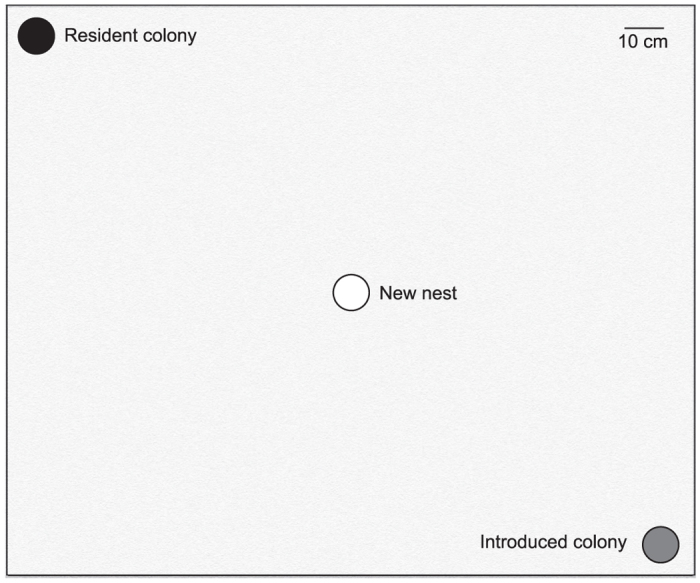
Schematic of the laboratory arena. This schematic diagram represents the laboratory arena (1.75 m × 1.45 m) used during the brood theft experiment. The black, grey and white circles represent the resident colony, the introduced colony and the empty nest, respectively. The empty nest was positioned at the centre, and the other two were positioned at randomly selected corners of the arena. The nests and the arena are drawn to scale.

**Figure 2:**
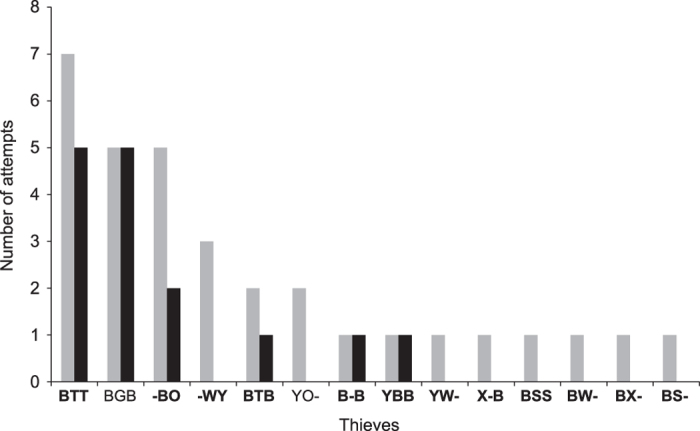
Attempts of brood theft by individual thieves. Number of attempts of brood theft by individual thieves across the eight replicates of the laboratory-based brood theft experiment, represented using bar diagram. A grey bar represents total number of attempts made by an individual thief, and the corresponding black bar represents the number of attempts by the same individual that were successful. The colour codes used for marking the thieves are given in the X-axis, identities in bold being thieves from resident colonies while others are from the introduced colonies.

**Figure 3:**
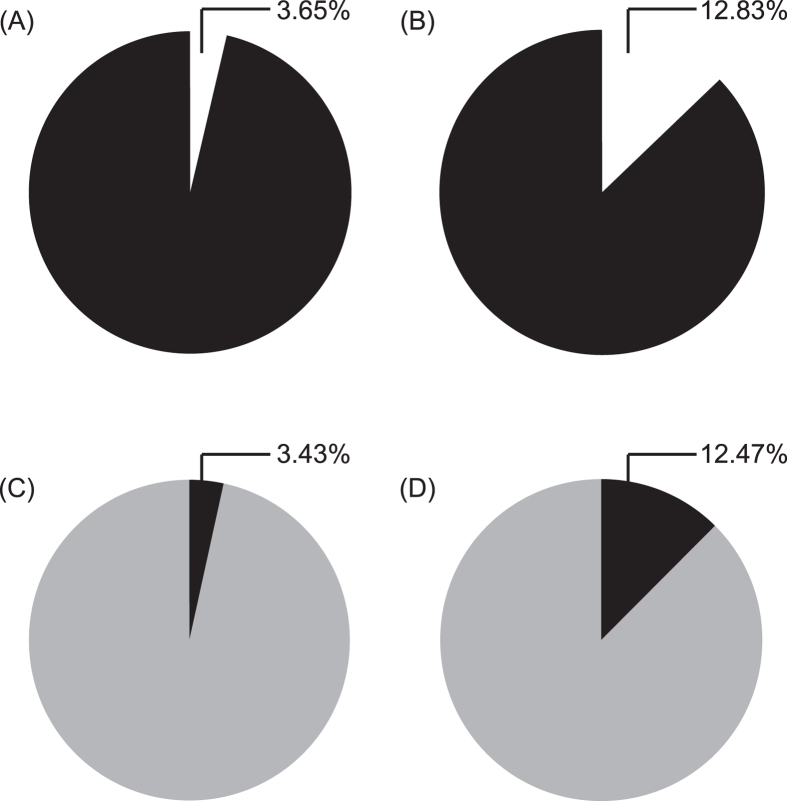
Loss and gain of brood due to theft. Pie diagram representation of the percentage of brood items stolen during the laboratory-based brood theft experiment. (**A**) Mean percentage of brood lost by the victim colonies and (**B**) mean percentage of pupae lost by the victim colony, represented using the missing portion of the black pie. (**C**) Mean percentage of brood gained by the thief colonies and (**D**) mean percentage of pupae gained by the thief colonies, represented using the black portion of pie inserted into the grey pie.

In the 8 replicates performed in the laboratory arena (for a schematic representation see Fig. 1), the relocation of the introduced colony took 101.4 ± 24.8 (mean ± SD) minutes. The number of adult females in the resident and introduced colonies were comparable (Wilcoxon paired-sample test: n = 8, T = 11.5, p = 0.41), but the number of attempts to steal brood by the two colonies were not. A total of 32 attempts of brood theft was observed. Resident colonies, which were undisturbed, made a total of 25 attempts in 6 replicates; whereas introduced colonies, which were more vulnerable as they were unsheltered and in the process of relocating to a new nest, made a total of 7 attempts in 2 replicates, which was significantly less than the former (Goodness of fit: χ^2^ = 10.1, df = 1, p < 0.01). A total of 15 attempts were successful, i.e. thieves were able to procure brood from a foreign colony and bring it back to its own nest. All the brood items were stolen from inside victim colonies’ nests – 14 items were stolen from old nests, and 1 item was stolen from new nest. No item was observed to be stolen from workers transporting brood during relocation. Of the successful stealing events, 10 were conducted by thieves from the resident colonies, and the rest of the 5 were by thieves from the introduced colonies. Even though introduced colonies attempted and were successful at stealing, the resident colonies seemed to be at an advantage as they made significantly more number of attempts and were successful more number of times compared to the introduced colonies. In 14 out of the 15 cases of theft pupae were stolen, and in only one case a larva was stolen. The percentage of thieves were not significantly different in the resident colonies (1.1 ± 0.9%) compared to the introduced colonies (0.3 ± 0.5%) (Wilcoxon paired-sample test: n = 8, T = 4, p = 0.09), and only 1.4 ± 0.7% adult females of a colony were involved in stealing. Individual thieves made 2.3 ± 2 attempts, of which 1.1 ± 1.8 were successful (Fig. 2). When considering the total brood of a thieving colony, they gained 3.4 ± 2.6% by stealing (Fig. 3C). As pupae were the most stolen item, we calculated the percentage gain in pupae. The thieving colonies added 12.5 ± 9.6% to their pool of pupae (Fig. 3D). The victim colonies were able to stop 53.1% of the total attempts. The primary mode of defence of the victim colony was to interact aggressively with the thieves. We observed 5 different types of aggressive interactions – antennal boxing, chasing, biting, dragging and holding down. Considering the total brood of a victim colony, they lost 3.6 ± 1.9% of it due to stealing (Fig. 3A); but considering only the total pupae, they lost 12.8 ± 3.8% of it (Fig. 3B).

### Brood theft in natural habitat

**Figure 4:**
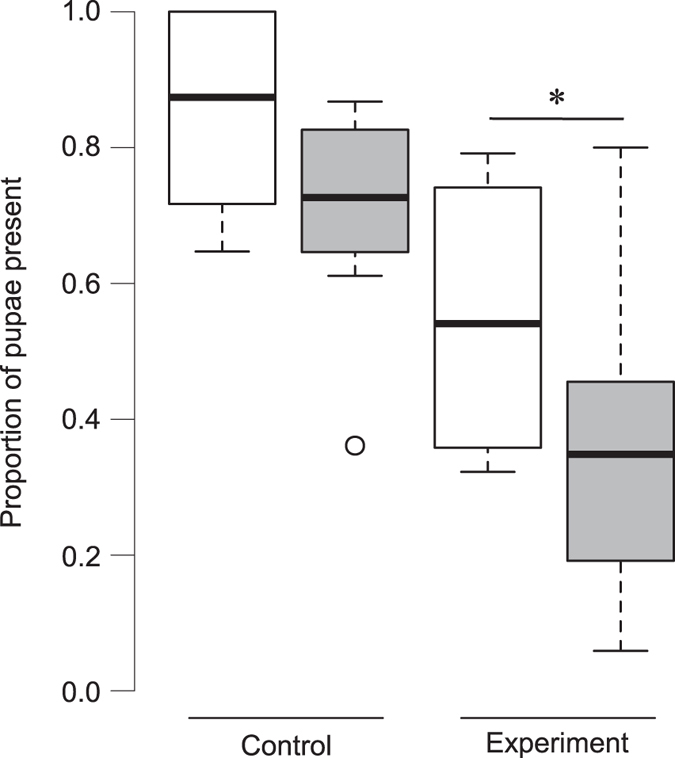
Indirect observation of brood theft in natural habitat. Comparison of expected and observed number of pupae in recollected colonies in the control and in the experiment of brood theft in natural habitat, represented using box and whisker plot. White boxes represent expected numbers and grey boxes represent observed numbers. The bold line inside the box represents the median and upper and lower walls of the box represents 1st and 3rd quartiles. The whiskers represent data points that are within 1.5 times the interquartile range, and outliers are represented by open circles. Observed and expected number of pupae (white and grey boxes, respectively) in control and experiment were compared using Wilcoxon paired-sample test (p < 0.05), and boxes that are significantly different are represented using an asterisk.

In the experimental colonies that were released in the natural habitat stealing events were recorded on the basis of *ad libitum* observations. Number of direct observations of stealing events was very low. During the 8 replicates of the experiment only 2 pupa were stolen from the released colonies by the members of conspecific colonies present in the vicinity of the release site. In the control, as there were no neighbouring colonies within the arena, no brood stealing was observed. All the colonies were collected back and the number of adults and pupae were counted to investigate the possibility of stealing in a 24 hour window as colonies settled themselves at their new nest. All of the recollected colonies had their gamergate, together with 93.1 ± 7.8% and 87.3 ± 12.8% (mean ± SD) adults of the colonies from control and experiment respectively. Therefore we presume that we were able to collect the whole colonies except the foragers that were outside. In the control, the expected number of pupae (28.3 ± 16.4) in the colonies after recollection was comparable to the observed number (24.4 ± 16.5) (Wilcoxon paired-sample test: T = 5, n = 8, p = 0.08); but in the experiment, the observed number of pupae (11.9 ± 10.4) was significantly lower than the expected number (17.3 ± 10.9) (Wilcoxon paired-sample test: T = 3, n = 8, p = 0.04) (Fig. 4) (see Supplementary Table S1). This indicates the possibility that in the presence of conspecific *D. indicum* colonies the experimental colonies lost 37.1 ± 30.9% of their pupae.

### Outcome of procured pupae

In the experiment to observe the fate of foreign pupae, the colonies were allowed to procure self-marked and foreign-marked pupae from the brood plate. The ants that procured this brood did not show any significant preference for foreign or self pupae (Mann-Whitney U test: U = 6249, df1 = 111, df2 = 111, p = 0.85). The procured pupae were brought back to the nest and either placed it in the brood pile of the colony or handed it over to a nestmate, who in turn placed the pupae in the brood pile. We examined the outcomes of a total of 371 pupae across the 8 replicates. This included self-unmarked, self-marked and foreign-marked categories, each in roughly equal proportions. On observing the colonies for 7 days we found that 262 pupae or 70.6% of pupae eclosed over this timespan. A total of 13 stillborn (incompletely developed and dead pupae) were found, i.e. 3.5% of the pupae died. No callow ant was found dead, all of them were inside the nest and within the cluster of nestmates. Throughout the observations, none of the pupae were seen to be consumed by the adults of the colony, thus we conclude that death or consumption of the pupae was not a major factor in our experiments. Further qualitative observations indicated that all callows were integrated into the colony. Not only they were present together with other adults, they even participated in caring for brood of the colony. Workers generally pick up brood and hold them in their mandibles when nests are disturbed, and 7 callow ants were seen holding brood when the colony received a mild disturbance during observation. The eclosion of pupae occurred simultaneously in more than one category, making it impossible to distinguish the callow ants based on the categories, therefore an overall count was taken for the aforementioned observations.

**Figure 5:**
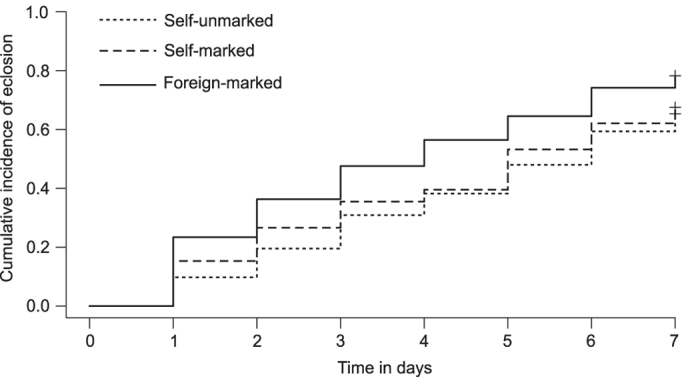
Ecosion of pupae of different categories across 7 days. The survival curves of the three pupae categories self-unmarked (dotted line), self-marked (dashed line) and foreign-marked (solid line) are plotted. The cumulative incidence of mortality, i.e. the probability of eclosion of the pupae is plotted in the Y-axis across seven days. Eclosion of the pupae in the three groups were compared using log-rank test and Cox proportional hazards regression model, and the rate of eclosion of the foreign-marked pupae were found to be higher compared to self-unmarked and self-marked categories (p < 0.05).

The eclosion of the three categories of pupae across seven days (see Supplementary Table S2) were compared using survival analysis, which is commonly used to assess and compare the survival of different study systems^25^,^26^,^27^. For the analysis, the eclosion of the pupae were considered as events, thus the probability of not eclosing was the survival probability. The survival of the three categories of pupae were found to be significantly different (Log-rank test: χ^2^ = 9.6, df = 2, p < 0.01). To understand which specific category of pupae caused the difference, we constructed a Cox proportionate hazard model of the three categories. According to the model, the survival of self-marked and self-unmarked pupae were comparable but the survival of foreign-marked pupae was significantly different with p < 0.05. To elaborate, the probability of eclosion of the self-unmarked and the self-marked categories were not significantly different (p = 0.87), but the probability of eclosion of foreign-marked category was 1.5 times higher and significantly different than that of self-unmarked (p = 0.01) (Table 1). Survival curves of the three pupae categories are given in Fig. 5. As the pupae are not dying by eclosing but rather are giving rise to adults, we have plotted the cumulative incidence of eclosion in the Y-axis.

## Discussion

Brood theft has been reported previously in ant subfamilies from temperate regions. In this study we report brood theft in a primitively eusocial ant *Diacamma indicum* belonging to the subfamily Ponerinae from the tropics. In the current study, we designed experiments to examine if *D. indicum* procure non-self brood and explicitly checked if they procure non-self, guarded brood from conspecific colonies, i.e. steal brood. We used non-neighbouring colonies to observe brood theft. Probability of multiple nests belonging to the same colony is low as *Diacamma* spp. are monodomous^20^ and they generally occupy pre-existing cavities^18^,^21^. Neighbouring nests may also not be closely related to each other as the colonies relocate frequently and outbreeding is suggested to be common^20^. In spite of these suggestions we paired only non-neighbouring colonies to eliminate the chance of them being recently fissioned from the same parent colony.

We see that *D. indicum* steals brood in lab conditions, and also possibly in their natural habitat. As the brood theft was done by individual ants bringing back single brood items and not by a coordinated team of ants conducting a large scale retrieval of items, we termed these observations as opportunistic theft as opposed to raids. Occurrence and frequency of theft is impacted by vulnerability of the colonies. Relocating colonies are particularly prone to stealing attempts by conspecific adults. The resident undisturbed colonies faced less number of brood stealing attempts; whereas the introduced colonies, who were exposed and in the process of relocation, faced significantly higher stealing attempts. Introduced colonies attempted to steal brood in only 2 replicates, and in each replicate there was only 1 adult who acted as a thief. Even though it seems that resident colonies had advantages which allowed them to make higher numbers of attempt to steal brood, it would not be meaningful to make quantitative comparison between the resident and introduced colonies in terms of success and failures of stealing attempts and risk and gain associated with brood theft with the current relatively small dataset.

The brood item of preference for theft was pupae, as among the 15 items stolen 14 were pupae. Thieving colonies gained on average 12.5% pupae, with 1.4% of adults involved in thievery. Defence against brood theft was present in the colonies; on average 33% of the total number of attempts by a colony were successful. We observed aggressive interactions towards the thieves, therefore the victim colony members recognized the thieves as non-nestmates and actively tried to defend their colony. Most of the brood items were stolen from inside the exposed old nest of the victim colony and it is unlikely for thieves to presume that they are retrieving their own colony’s brood as they would be surrounded by a foreign gestalt and foreign ants. As pupae were preferred during stealing, further experiments in this study dealt only with pupae as a brood item. The resident and introduced colonies were not significantly different in terms of number of adults, thus additional experiments with unequal colony sizes are required to understand the effect of number of adults and brood in colonies on brood theft.

Brood theft was observed in nature, and thus is not an artefact of laboratory conditions. We directly observed only two brood stealing events in nature. This was very low compared to laboratory-based experiments, and may not be reflective of the true magnitude of brood theft in nature. The reason for this could be the difficulty of observations. The complex terrain composed of grasses, weeds, leaves, rocks etc. in natural habitat made observation of events difficult and possibly reduced the encounter of non-nestmates. Further, the laboratory arena may have impacted the process of brood theft and defence against theft, resulting in a higher estimate of brood theft. Only further investigations in both natural and laboratory conditions will give us an accurate picture of brood theft in this species. However, brood theft could have occured in these re-establishing colonies following the 6 hours of observation period. In order to check for theft in the next 24 hours we collected the colonies back and counted the number of pupae present. This observed number of pupae was compared to the expected number of pupae in order to check for theft. We found that the former was significantly less than the latter in the experiment but not in the control, which suggests that pupae were possibly stolen from the focal colony by the neighbouring colonies. The environmental and physical factors that can cause a decrease in the number of pupae such as death, increased eclosion of pupae due to stress during relocation and loss of pupae during relocation were similar in control and experiment. The only difference in the setup of the experiment was the presence of neighbouring conspecific colonies indicating that the loss of pupae from the focal colony is possibly due to thievery, but to prove the claim further experiments are required to investigate the neighbouring nests for presence of stolen brood.

Brood theft has been recorded in ants for the purpose of slave-making, colony-foundation and consumption. For investigating the purpose of opportunistic theft in *D. indicum*, we observed the fate of procured foreign pupae in colonies. To rule out the effects of the process of procurement, if any, on the treatment towards the pupae, we allowed the colony to procure foreign pupae (foreign-marked) as well as their own (self-marked) by placing them together in a common brood pile. Further, the presence of a second control in the form of undisturbed and unmarked self-pupae in the colonies allowed us to contrast the impact of marking and disturbance we caused as well. Adults did not differentiate between self and foreign pupae during procurement, collected both without any preference and carried it back to the nest. After the pupae were brought back to the nest, the treatment towards the foreign pupae were comparable to their own pupae. During the seven days of qualitative observation, treatment by the adults of the colony towards all the three categories of pupae and the callow ants that eclosed from them was similar, though there was a difference in the rate of eclosion across the categories as the foreign pupae eclosed at a faster rate than the self pupae (both self-marked and self-unmarked categories). Marking, handling and procurement *per se* did not impact the probability of eclosion as self-unmarked and self-marked pupae eclosed at similar rates. The pupae were chosen from the colonies randomly, thus the age of the pupae is not likely to cause the faster rate of eclosion for the foreign pupae, but additional experiments are necessary to find out the underlying cause. Apart from the differences in the rate of eclosion, the treatment and behaviour of the adult members of the colonies towards the newly eclosed ants were qualitatively similar, and whether they eclosed from the self-pupae or the foreign-pupae, they were present together with other adults inside the colonies. Therefore, the foreign pupae were not procured for consumption, and the observations suggest that they were possibly procured to increase the workforce of the colonies. It is interesting that pupae was the preferred brood item to be stolen as they will directly eclose as adult ants without requiring any additional investment from the thief colony, and thus are the most advantageous to steal.

Most of the previous reports of brood theft have been from temperate regions, and mostly on species of ants belonging to the subfamilies Myrmicinae and Formicinae. The current study is important because it explores the aspect of brood theft in a primitively eusocial ant *Diacamma indicum*, belonging to the Ponerinae subfamily of the largely unexplored poneromorph group of subfamilies^28^. In this study we propose a new dimension to the costs of relocation – vulnerability of brood to theft by conspecific neighbours. From our laboratory experiments as well as experiments in the natural habitat we see that increased vulnerability of colonies during relocation may enhance the chances of brood theft. Additional experiments in the natural habitat investigating brood theft in newly established and well established colonies are required to investigate the extent of this phenomenon. In this species theft of brood is opportunistic, which can be a possible origin for more organized raiding behaviour. The procured brood is not consumed in *D. indicum*, rather the conspecific pupae are allowed to eclose and the callow ants integrate into the thieving colony. In addition to behavioural observations, use of genetic markers to assess relatedness among nestmates^29^ can be a useful method to investigate occurrence of brood theft and the fate of the procured brood. For example, in a study in the harvester ant *Pogonomyrmex rugosus* foreign workers were found in several colonies using genetic analysis, and the occurrence of brood raiding was suggested to be the probable cause^30^. Examination of brood procurement and stealing in other species belonging to subfamily Ponerinae and other primitively eusocial ant species will provide a framework to understand the origin and evolution of brood theft.

## Methods

*D. indicum* colonies were collected from Mohanpur, Nadia, West Bengal, India (22°56′ N, 88°31′ E) using the nest flooding technique, where the colonies are driven out from their natural nest by a steady flow of water. Colony members that walk out of the flooded nest enter a plastic tube that is connected to a dark plastic container that acts as a temporary shelter^19^. Colonies thus collected were brought back to the laboratory and housed in plastic boxes (28.5 cm × 21.5 cm × 12 cm) with plaster of Paris base. Presence of gamergate was confirmed by inspecting all adult females for the presence of gemmae. Only colonies that had a female with its gemmae intact (gamergate) were used for the experiments. Colonies were provided with *ad libitum* food^31^, water and occasionally termites. Experiments were performed with intact colonies soon after collection so that the number of adults and brood are reflective of natural colonies. Adult members, larvae and pupae of the colonies were marked with combinations of enamel paint colours (Testors, Rockford, IL, USA) for colony-level as well as individual identification as required by the experiment. As all adult females in the colonies are monomorphic, and one of the workers become the reproductive individual, i.e. gamergate, there is no morphological difference between worker and reproductive destined brood^20^,^22^,^32^. The only morphological difference is between male and females^33^, but the brood of these two categories are indistinguishable by visual examination (personal observation), thus we treat all brood as belonging to only one category. Experiments in the laboratory were performed in a sand arena of 1.45 m × 1.75 m area (laboratory arena). Control experiments in the field were performed inside a field arena. This arena was built by enclosing 1.25 m × 1.55 m area in the natural habitat of *D. indicum* by sinking in Plexiglas boards into the soil. The surface of these Plexiglas walls were coated with petroleum jelly to ensure that the focal colony did not escape from the arena, and special care was taken to ensure that other *D. indicum* colonies had not occupied the arena. Neither the substrate nor the vegetation inside the arena was disturbed to ensure that it resembles the natural environment of the ants.

### Brood theft in laboratory

We performed experiments in the laboratory to observe if *D. indicum* has the tendency to steal brood from conspecific colonies. Brood theft is defined as the procurement of brood from a foreign colony that is guarded by adults. We used 16 colonies to perform 8 replicates during July-September 2014. Colonies consisted of 130.9 ± 40.5 (mean ± SD) adult females, 33.3 ± 17.6 pupae, 24.1 ± 12.5 larvae and 42.5 ± 17.9 eggs. Two colonies were used simultaneously for each replicate. The pair of colonies used for a given replicate were collected from non-neighbouring nests to ensure that they were not part of the same colony or were not recently fissioned from the same parent colony. All members of a colony received one common colour dot on their body for colony identification. In addition each ant received a combination of other colours to enable us to identify individual ants in a unique manner. Before the start of the experiment, one of the colonies was placed at a randomly chosen corner of the arena for a minimum of twelve hours to allow the colony to familiarize with the arena. This colony was referred to as the resident colony. At the start of the experiment a second colony was placed at another randomly chosen corner of the arena, and was referred to as the introduced colony. The introduced colony was made to relocate by removing the top cover of the nest, placing a light source directly above the nest and providing an empty nest at the centre of the arena. The resident colony did not face any disturbance (for a schematic representation see Fig. 1). The distance between the old and the new nest for the relocating introduced colony was 1.13 m, which was within the range of distance between old and new nests occupied by relocating colonies in nature^24^. The distance between the introduced and the resident colony was the length of a diagonal of the square arena, i.e. 2.27 m. We do observe neighbouring nests of *D. indicum* in nature which are closer than 2.27 m (personal observation), therefore the setup in the arena was realistic. In previous experiments on *D. indicum* in their natural habitat, we have observed that unfamiliarity of the site does not impact the relocation dynamics as the time taken for relocation, the number of temporary sites occupied and the distance between the old and the final nest were similar when the relocation was done in an unfamiliar site compared to relocation in a familiar site^24^. Therefore we did not expect unfamiliarity of the relocation sites to be a critical factor impacting the relocation process in our experiments.

Interactions between members of the two colonies and any attempt to steal brood were recorded. All tandem runs conducted by the introduced colony in the process of relocation into the new nest was also recorded. All recordings were done by placing two video cameras directly above the nests of the resident and introduced colonies, and events occurring outside the nests were recorded using a voice recorder. The experiment was terminated one hour after the introduced colony relocated into the new nest. The relocation was considered complete when all the brood and all the colony members (except tandem leaders) had evacuated the old nest. Ants were said to attempt to steal brood when they partially or fully picked up a brood item that belonged to the non-self colony in their mandible, and ants that attempted to steal were termed as thieves. An attempt to steal was scored as successful if the thief succeeded in carrying the brood item from the foreign nest back to its own nest (see Supplementary Video S3), else the attempt was scored as unsuccessful (see Supplementary Video S4). The resident and the introduced colonies were compared in terms of the number of successful and unsuccessful stealing attempts. The impact of vulnerability caused due to relocation in the introduced colony was compared to undisturbed resident colonies in terms of number of thieves, the number of attempts to steal brood, the loss and/or gain of brood, the rate of success and the potential risk faced by the thieves.

### Brood theft in natural habitat

A field experiment was performed to observe the prevalence of brood theft in their natural habitat. We used two approaches to address this issue – direct observation of theft over a short time period and collection of indirect evidence of theft over relatively longer time period. Both of these approaches are explained below.

### Direct observation of theft

Eight replicates were performed using eight colonies during March-June 2014, consisting of 111 ± 43.5 (mean ± SD) adult females, 30.2 ± 11.2 pupae, 22.4 ± 13.1 larvae and 42.9 ± 19.7 eggs. The experiment was conducted across a span of four days. Colonies were collected on the first day and the number of adult members and pupae were counted. On the second day, adults and pupae were marked with a single colour to identify them separately from other colonies present in the natural habitat. On the third day, the colonies were released in the field in the vicinity of other conspecific nests. When *D. indicum* colonies relocate in natural habitat, they move to temporary nests located at a distance of 1 m on average from the release site before they move to a final nest^22^. Therefore, in the current experiment we released the marked colony within 1 m radius of other conspecific nests. Further, this allowed us to ensure that the selected habitat was amiable for *D. indicum*. In order to examine stealing events directly, *ad libitum* observation was conducted for 6 hours − 10 am to 4 pm. All aggressive interactions between non-nestmates, attempt of entry into foreign colony and attempt of brood theft was recorded.

### Indirect evidence of theft

In addition to trying to directly observe theft, we also collected indirect proof of theft. The colonies were collected back on the fourth day using the nest flooding technique described previously, and all the adults and pupae were counted. The number of pupae present upon recollection on the fourth day is expected to be dependent on the initial number of pupae, the number of larvae that pupate and the number of adults that eclose. The rate of pupation was observed to be very low across day 1 to 3 and thus this factor was omitted for further calculations. Therefore, the number of pupae present in the recollected colonies was predicted using the initial number of pupae and the number of eclosions. If the number of pupae on *x*th day is *P*_*x*_ and on the previous day is *P*_*x−1*_, then the proportion of pupae present on *x*th day (*R*_*x*_) is *P*_*x*_/*P*_*x*−*1*_, and therefore the expected number of pupae on (*x* + *1*)th day (*E*_*x*+*1*_) can be calculated using the following equation:


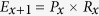


The values for *P*_*x*−*1*_ and *P*_*x*_ was collected for each colony while they were in the laboratory on the first and second day respectively, and was used to calculate the expected number of pupae (*E*_*x*+*1*_) on the fourth day in the recollected colony using the equation mentioned above. The expected number was compared with the observed number of pupae. If the observed number of pupae on the fourth day was lower than the expected number then we conclude that pupae are missing from the colony. If observed pupae number was higher than expected and if those pupae were unmarked then it would indicate that the focal colonies had stolen pupae from neighbouring colonies. Further, the presence of unmarked callow would allow us to elucidate the number of pupae that have eclosed.

A control experiment was performed in the field arena to control for several factors other than stealing which can impact the number of pupae present in the recollected colonies, such as loss due to misplacement during relocation, death, disturbance caused by flooding etc. This experiment was conducted in the field arena which was free of interferences from other *D. indicum* colonies. This prevented any brood stealing by conspecific colonies ensuring that the change in number of pupae were only due to stress caused by processing and relocation during the experiment. The protocol for the control experiment was similar to the field experiment, the only difference being the use of the field arena as the release site of the colony on the third day. Eight replicates were done using eight colonies collected during May-August 2015, consisting of 146.6 ± 52.7 adult females, 34.4 ± 18.5 pupae, 29.5 ± 10.7 larvae and 55.4 ± 22 eggs. The number of pupae present in the recollected colonies on the fourth day was compared to the expected number of pupae that was calculated using the method described in the previous paragraph.

### Outcome of procured pupae

A laboratory-based experiment was performed to observe the outcome of foreign pupae that were procured by the colonies. We performed this experiment to address whether the procured brood is consumed or allowed to eclose and become slaves. Eight replicates were performed using eight colonies during March-June 2015, consisting of 95.6 ± 29.4 (mean ± SD) adult females, 35.3 ± 18 pupae, 18.4 ± 9 larvae and 39.9 ± 22.7 eggs. The experiment was performed across nine days. After collection of colonies, the number of adults and brood were counted on the first day. On the second day, the adult females were marked uniquely using combination of paints. Half of the pupae present in the colony were randomly picked and marked with a single colour and were referred to as self-marked pupae. The other half were left unmarked and were referred to as self-unmarked pupae. The self-marked pupae were kept separate for a minimum of six hours, along with half their number of adult females for tending them. These females were chosen only from those individuals who were holding a pupae during the time of marking and thus were familiar with the brood. Pupae from a different colony were picked randomly and were marked with a different colour (foreign-marked pupae). The self-marked pupae, together with same number of foreign-marked pupae, were placed at a random corner of the arena. The colony was placed at another random corner without causing any disturbance, and the manner in which the pupae were discovered, handled and brought back to the nest was recorded using a video camera until the last brood item was removed from this site. Ranks were given to the brood items according to the order in which they were brought back by the ants, and later were analysed to check if there was any preference for self-pupae or foreign pupae during procurement. After all the pupae were taken, the previously separated members of the colony were returned. Observation of the fate of the pupae of the three categories i.e. self-unmarked, self-marked and foreign-marked were conducted for the next seven days. The observation was done twice a day to note the location of the pupae in the nest, the total number of newly eclosed adults, the number of newly eclosed adults from each category of pupae and their activity. During the observations care was taken to cause as little disturbance as possible. All the newly eclosed adults were marked with colour combination according to the day of eclosion. However, all newly eclosed adults could not be assigned unambiguously to its category, especially when more than one adult had eclosed before an observation. Thus, the number of intact pupae in each category was recorded and compared. The daily observations were used to assess if the self and foreign pupae and callow ants are treated differently by the colony.

Throughout the three sets of experiments behavioural observations were recorded using either video cameras, voice recorders or manually. Data was decoded from these recordings or were directly entered into observation sheets and was later entered into spreadsheets for further analysis. Non-parametric and semi-parametric tests were used to test the hypothesis and two-tailed values of p < 0.05 was used as the cut-off to accept the alternative hypothesis. Unless mentioned otherwise mean ± standard deviation values are reported. Statistical tests were done using statistiXL 1.10 and R 3.1.0.

## Additional Information

**How to cite this article**: Paul, B. *et al*. Opportunistic brood theft in the context of colony relocation in an Indian queenless ant. *Sci. Rep.* **6**, 36166; doi: 10.1038/srep36166 (2016).

**Publisher’s note:** Springer Nature remains neutral with regard to jurisdictional claims in published maps and institutional affiliations.

## Supplementary Material

Supplementary Information

Supplementary Video S3

Supplementary Video S4

## Figures and Tables

**Table 1:** Result of Cox proportionate hazard test for comparing the “survival” of pupae, i.e. proportion of pupae that remained as pupae and did not eclose as an adult over the given time span of the three categories - self-unmarked, self-marked and foreign-marked.

Covariate	Hazard Ratio	Lower CL	Upper CL	p
Self-marked	1.03	0.76	1.39	0.87
Foreign-marked	1.50	1.12	2.01	0.01

The hazard ratio depicts how many times the probability of survival of the covariates are compared to self-unmarked. The lower and upper CL (CL = confidence limit) gives the confidence interval of the hazard ratio.
