# Supplementary material for: Opportunistic brood theft in the context of colony relocation in an Indian queenless ant
Source: Sci Rep. 2016 Oct 31;6:36166. doi: 10.1038/srep36166 (PMC5087078; doi:10.1038/srep36166)
Supplement: Supplementary Information [file srep36166-s1.pdf]

Opportunistic brood theft in the context of colony relocation in an Indian queenless ant

Bishwarup Paul[1], Manabi Paul[1] and Sumana Annagiri[1*]

Supplementary information

[1*]Address for correspondence:
Behaviour & Ecology Lab, Department of Biological Sciences, Indian Institute of Science Education and Research, Kolkata, Mohanpur, West Bengal 741246, India
Email: sumana@iiserkol.ac.in
Phone: 91-33-25873017
Fax: 91-33-25873028

Abbreviated title: Brood theft in an Indian queenless ant

Supplementary information 1

Supplementary Table S1

Replicate	Control					Experiment				
	Pupae number observed			Pupae number expected		Pupae number observed			Pupae number expected	
	Day 1	Day 2	Day 4	Day 3	Day 4	Day 1	Day 2	Day 4	Day 3	Day 4
1	36	33	13	30.25	27.73	21	15	8	10.71	7.65
2	40	37	30	34.22	31.66	17	12	1	8.47	5.98
3	37	32	26	27.67	23.93	47	43	23	39.34	35.99
4	47	41	32	35.77	31.20	19	17	8	15.21	13.61
5	68	67	59	66.01	65.04	38	28	12	20.63	15.20
6	15	15	13	15	15	25	22	6	19.36	17.04
7	14	14	11	14	14	35	24	5	16.46	11.28
8	18	18	11	18	18	40	37	32	34.22	31.66

Supplementary Table S1: Observed and expected number of pupae in the colonies of the experiment for brood theft in natural habitat are presented. The number of pupae across the 4 days of experiment for the control and the experiment set are given. Observed number of pupae in the colonies are given for days 1, 2 and 4. Expected number of pupae in the colonies are given for days 3 and 4, which were calculated using the observed number of pupae on days 1 and 2 as described in the methods section.

Supplementary information 2

Supplementary Table S2

**Self-unmarked pupae**								
**Day \ Replicate**	**1**	**2**	**3**	**4**	**5**	**6**	**7**	**8**
**0**	12	11	17	11	16	37	12	7
**1**	12	9	17	9	16	32	12	5
**2**	12	8	14	9	14	30	10	3
**3**	9	5	14	9	12	26	9	2
**4**	6	5	13	8	12	23	9	1
**5**	5	4	11	6	12	20	7	0
**6**	4	1	11	6	9	17	3	0
**7**	3	0	9	5	8	14	1	0
**Self-marked pupae**								
**Day \ Replicate**	**1**	**2**	**3**	**4**	**5**	**6**	**7**	**8**
**0**	12	11	17	11	16	37	12	8
**1**	12	6	17	9	12	34	9	6
**2**	10	6	16	8	9	28	9	5
**3**	9	4	15	5	7	26	9	5
**4**	7	4	15	5	6	25	8	5
**5**	7	2	13	5	6	13	8	4
**6**	5	1	11	5	6	10	7	2
**7**	5	1	10	4	6	9	6	2
**Foreign-marked pupae**								
**Day \ Replicate**	**1**	**2**	**3**	**4**	**5**	**6**	**7**	**8**
**0**	12	11	17	11	16	37	12	8
**1**	12	7	17	8	10	27	8	7
**2**	12	7	15	7	5	24	3	7
**3**	7	4	15	7	4	21	1	6
**4**	7	3	12	6	4	18	1	3
**5**	6	1	11	6	4	12	1	3
**6**	6	0	8	4	2	8	1	3
**7**	6	0	7	3	2	5	1	3

Supplementary Table S2: Eclosion of pupae in the colonies of the experiment for observing the outcome of the procured pupae is represented. The table lists the number of pupae of the three categories – self-unmarked, self-marked and foreign-marked present in the 8 colonies used in the experiment across the 7 days of observation.

Supplementary information 3

Supplementary Video S3 – Pupae stealing: Successful stealing event of pupae by a thief ant from a victim colony. All the ants of the victim colony are marked with golden as the common colour, whereas all the ants of the thieving colony have green as the common colour. The thieving ant (marked green-blue-blue or GBB) steals a pupae from the victim colony, returns to its own colony and hands over the stolen pupae to a nestmate.

Supplementary information 4

Supplementary Video S4 – Unsuccessful attempt to steal: Unsuccessful stealing attempt of pupae by a thief ant from a victim colony. All the ants of the victim colony are marked with green as the common colour, whereas all the ants of the thieving colony are marked with golden as the common colour. The thieving ant (marked yellow-golden-golden or YXX) tries to steal a pupae from the victim colony, but gets caught and stopped by members of the victim colony.
